# The Efficacy and Safety of Tandem Transplant Versus Single Stem Cell Transplant for Multiple Myeloma Patients: A Systematic Review and Meta-Analysis

**DOI:** 10.3390/diagnostics14101030

**Published:** 2024-05-16

**Authors:** Yu-Han Chen, Lindsay Fogel, Andrea Yue-En Sun, Chieh Yang, Rushin Patel, Wei-Cheng Chang, Po-Huang Chen, Hong-Jie Jhou, Yeu-Chin Chen, Ming-Shen Dai, Cho-Hao Lee

**Affiliations:** 1 Department of Internal Medicine, Englewood Hospital and Medical Center, Englewood, NJ 07631, USA; yuhanchen002@gmail.com; 2 Hackensack Meridian School of Medicine, Nutley, NJ 07110, USA; lindsay.fogel@hmhn.org; 3 College of Medicine, National Yang Ming Chiao Tung University, Taipei 112, Taiwan; andreaysun@gmail.com; 4 Department of Internal Medicine, School of Medicine, University of California Riverside, Riverside, CA 92521, USA; 5 Department of Internal Medicine, Community Hospital of San Bernardino, San Bernardino, CA 92411, USA; rushinpateldr@gmail.com; 6 Department of Ophthalmology, Taoyuan General Hospital, Ministry of Health and Welfare, Taoyuan 330, Taiwan; cwc761229@gmail.com; 7 Department of Internal Medicine, Tri-Service General Hospital, National Defense Medical Center, Taipei 114, Taiwan; chenpohuang@hotmail.com; 8 Department of Neurology, Changhua Christian Hospital, Changhua 500, Taiwan; xsai4295@gmail.com; 9 Division of Hematology and Oncology Medicine, Department of Internal Medicine, Tri-Service General Hospital, National Defense Medical Center, Taipei 114, Taiwan; yeuchin99@gmail.com (Y.-C.C.); dms1201@gmail.com (M.-S.D.)

**Keywords:** tandem autologous transplantation, autologous stem cell transplantation, multiple myeloma, meta-analysis

## Abstract

**Background**: While high-dose therapy and autologous stem cell transplant (ASCT) remain integral to the primary treatment of newly diagnosed transplant-eligible multiple myeloma (MM) patients, the challenge of disease progression persists. The primary objective of this meta-analysis is to evaluate the efficacy and safety of tandem ASCT compared to single ASCT. **Methods**: We conducted a systematic review and meta-analysis of randomized controlled trials and observational studies comparing tandem ASCT with single ASCT in patients with newly diagnosed MM. We searched PubMed, EMBASE, Cochrane Library, and Clinical Trials databases for studies published up to January 2024. The primary outcomes were progression-free survival (PFS), overall survival (OS), overall response rate (ORR), complete response rate (CRR), and treatment-related mortality (TRM). We used a random-effects model to calculate pooled hazard ratios (HRs) and odds ratios (ORs) with 95% confidence intervals (CIs). Study quality was assessed using the Cochrane Risk of Bias tool and Newcastle-Ottawa Scale. **Results**: Eleven studies involving 4862 patients met the inclusion criteria. Tandem ASCT was associated with a significantly higher CRR compared to single ASCT (OR 1.34, 95% CI 1.10–1.63, I^2^ = 31%), but no significant differences were observed in PFS (HR 0.76, 95% CI 0.44–1.33, I^2^ = 15%), OS (HR 0.63, 95% CI 0.36–1.09, I^2^ = 28%), or the ORR (OR 0.92, 95% CI 0.75–1.10, I^2^ = 31%). However, tandem ASCT was associated with a significantly higher risk of TRM (OR 2.36, 95% CI 1.76–3.16, I^2^ = 31%). **Conclusions**: Tandem ASCT improves CRR but does not provide significant benefits in terms of PFS, OS, or ORR compared to single ASCT in patients with newly diagnosed MM. Moreover, tandem ASCT is associated with a higher risk of TRM. The decision to pursue tandem ASCT should be made on an individual basis, carefully weighing the potential benefits and risks in light of each patient’s unique clinical situation. Future research should focus on identifying patient subgroups most likely to benefit from tandem ASCT and exploring strategies to optimize the efficacy and safety of this approach in the context of novel agent-based therapies.

## 1. Introduction

Multiple myeloma (MM) is a hematologic malignancy characterized by the clonal proliferation of plasma cells in the bone marrow, leading to the overproduction of monoclonal immunoglobulins, bone destruction, and other clinical manifestations [1]. MM accounts for approximately 1.8% of all new cancer cases and 2.1% of all cancer-related deaths in the United States [2]. The disease primarily affects older individuals, with a median age at diagnosis of 69 years [3]. Despite significant advancements in treatment options over the past two decades, MM remains an incurable disease, with most patients experiencing relapse and requiring subsequent lines of therapy [4].

The introduction of novel agents, such as proteasome inhibitors (e.g., bortezomib, carfilzomib), immunomodulatory drugs (e.g., lenalidomide, pomalidomide), and monoclonal antibodies (e.g., daratumumab, elotuzumab, and isatuximab), has revolutionized the treatment landscape of MM [5]. These agents have significantly improved response rates, progression-free survival (PFS), and overall survival (OS) when used in combination with conventional chemotherapy or as monotherapy [6]. However, high-dose chemotherapy followed by autologous stem cell transplantation (ASCT) remains a crucial component of the treatment algorithm for eligible patients with newly diagnosed MM [7,8].

ASCT has been shown to prolong PFS and OS compared to conventional chemotherapy alone in several randomized controlled trials [9]. The procedure involves the collection of the patient’s own hematopoietic stem cells, followed by the administration of high-dose chemotherapy (typically melphalan) to eradicate the malignant plasma cells. The previously collected stem cells are then infused back into the patient to reconstitute the bone marrow and hasten hematologic recovery [10]. While ASCT is considered a standard of care for transplant-eligible patients, the majority of patients will eventually relapse, with a median PFS of 2–4 years [11].

To further improve treatment outcomes, the concept of tandem ASCT has been explored in several studies. Tandem ASCT involves performing two sequential ASCTs within a short period (typically 4–6 months), with the goal of achieving deeper and more durable responses [12]. The rationale behind this approach is that a second round of high-dose chemotherapy and ASCT may eradicate residual myeloma cells that survived the first transplant, thereby prolonging remission duration and potentially improving survival [13].

However, the clinical benefit of tandem ASCT compared to single ASCT remains controversial. While some studies have reported improved response rates and survival outcomes with tandem ASCT [14], others have found no significant differences between the two approaches [15,16]. Additionally, tandem ASCT is associated with increased toxicity and higher treatment-related mortality (TRM) compared to single ASCT [17], raising concerns about its risk-benefit profile.

The heterogeneity in patient populations, treatment regimens, and study designs across trials investigating tandem ASCT in MM has made it challenging to draw definitive conclusions about its efficacy and safety. Some studies have suggested that certain subgroups of patients, such as those with high-risk cytogenetics or suboptimal response to initial therapy, may benefit more from tandem ASCT [18,19]. However, these findings have not been consistently replicated, and the optimal patient selection criteria for tandem ASCT remain unclear.

Furthermore, the rapidly evolving landscape of MM treatment, with the increasing use of novel agents and continuous therapy approaches, has raised questions about the role and timing of ASCT in the modern era [20]. Some experts have argued that the upfront use of tandem ASCT may not be necessary for all patients, given the availability of effective salvage options at relapse [21]. Others have proposed that tandem ASCT could be reserved for patients who fail to achieve a deep response after initial therapy or those with high-risk features [22].

Given the conflicting evidence and ongoing debates surrounding the use of tandem ASCT in MM, there is a pressing need for a comprehensive and updated systematic review and meta-analysis to synthesize the available data and provide evidence-based recommendations for clinical practice. While previous studies have been conducted on this topic, they have several limitations, such as the inclusion of a small number of trials, lack of subgroup analyses, and failure to account for the quality of included studies [23,24].

To address these gaps in the literature, we conducted a systematic review and meta-analysis of randomized controlled trials and observational studies comparing tandem ASCT with single ASCT in patients with newly diagnosed MM. Our primary objective was to evaluate the efficacy of tandem ASCT in terms of response rates, PFS, and OS. Secondary objectives included assessing the safety of tandem ASCT, particularly TRM, and exploring potential sources of heterogeneity across studies.

By providing a rigorous and up-to-date summary of the evidence on tandem ASCT in MM, our study aims to inform clinical decision-making, guide future research efforts, and ultimately improve outcomes for patients with this challenging disease. The findings of this meta-analysis will be of interest to hematologists, oncologists, transplant specialists, and other healthcare professionals involved in the care of MM patients, as well as researchers, policymakers, and patient advocates.

## 2. Materials and Methods

### 2.1. Search Strategy

A thorough review of existing literature was conducted to locate studies published up to January 2024. Primary sources included the PubMed, EMBASE, Cochrane Library, and Clinical Trials databases. Electronic searches were carried out using a search algorithm that combined MeSH terms, Emtree synonyms, and free words. The search strategy was designed to be comprehensive and sensitive, ensuring the identification of all relevant studies comparing tandem ASCT with single ASCT in MM patients.

In addition to the electronic database searches, we also employed supplementary search methods to minimize the risk of missing important studies. These methods included hand searching the reference lists of included studies and relevant review articles, as well as searching for grey literature sources, such as conference proceedings, abstracts, and unpublished trials. We also consulted with experts in the field to identify any ongoing or recently completed studies that may not have been captured by our search strategy.

There were no restrictions on the publication language of the studies, and translations were obtained when necessary. The search criteria focused specifically on studies comparing tandem ASCT and single ASCT in patients with newly diagnosed MM. Studies involving other plasma cell dyscrasias, such as amyloidosis or POEMS syndrome, were excluded.

The detailed search strategy, including the specific MeSH terms, Emtree synonyms, and free words used, is provided in the Supplementary Materials (S2). The search results were independently screened by two reviewers (CHL and WCC) to identify potentially eligible studies based on the title and abstract. The full texts of these studies were then retrieved and assessed for inclusion based on the predefined eligibility criteria.

### 2.2. Inclusion and Exclusion Criteria

The PRISMA (Preferred Reporting Items for Systematic Reviews and Meta-Analyses) checklist was used to guide the reporting of this meta-analysis (Supplementary Materials S1). Studies were eligible for inclusion if they met the following criteria: (1) randomized controlled trials or observational cohort studies comparing tandem ASCT with single ASCT in patients with newly diagnosed MM; (2) studies reporting at least one of the following outcomes: response rates (overall response rate (ORR), complete response rate (CRR)), PFS, OS, or TRM; (3) studies with a minimum follow-up duration of 12 months; and (4) studies published in peer-reviewed journals or presented as abstracts at major international conferences.

Studies were excluded if they met any of the following criteria: (1) non-comparative studies, such as case reports, case series, or single-arm trials; (2) studies involving patients with relapsed or refractory MM, or other plasma cell dyscrasias; (3) studies comparing tandem ASCT with allogeneic stem cell transplantation or other non-ASCT therapies; (4) studies with incomplete, retracted or duplicated data; and (5) animal studies, in vitro studies, or studies without original data (e.g., reviews, commentaries, editorials).

In cases of multiple publications from the same study population, only the most recent or comprehensive report was included to avoid duplication of data. If necessary, the authors of the original studies were contacted for clarification or to obtain additional data.

### 2.3. Data Extraction

Two reviewers (CHL and WCC) independently extracted data from the included studies using a standardized data collection form. The extracted information included study characteristics (first author, year of publication, study design, country, sample size, inclusion and exclusion criteria), patient characteristics (age, sex, disease stage, cytogenetic risk, prior therapies), treatment details (induction regimen, mobilization protocol, conditioning regimen, number of CD34+ cells infused, maintenance therapy), outcomes (ORR, CRR, VGPR, PFS, OS, TRM), follow-up duration, and funding sources.

Any discrepancies in the extracted data between the two reviewers were resolved through discussion and consensus. If a consensus could not be reached, a third reviewer (YHC) was consulted to make the final decision. The extracted data were then entered into a Microsoft Excel spreadsheet for further analysis.

### 2.4. Quality Assessment

The quality of the included studies was independently assessed by two reviewers (CHL and WCC) using the Cochrane Risk of Bias tool [25] for randomized controlled trials and the Newcastle-Ottawa Scale (NOS) [26] for observational studies. The Cochrane Risk of Bias tool evaluates the risk of bias across six domains: random sequence generation, allocation concealment, blinding of participants and personnel, blinding of outcome assessment, incomplete outcome data, and selective reporting. Each domain was judged as having a low, high, or unclear risk of bias based on the criteria specified in the Cochrane Handbook for Systematic Reviews of Interventions [27].

The NOS assesses the quality of observational studies based on three broad categories: selection of study groups, comparability of groups, and ascertainment of exposure or outcome. Studies were awarded stars in each category based on predefined criteria, with a maximum of four stars for selection, two stars for comparability, and three stars for exposure or outcome. Studies with a total score of seven or more stars were considered to be of high quality, while those with a score of five or six stars were considered to be of moderate quality, and those with a score of four or fewer stars were considered to be of low quality.

Any discrepancies in the quality assessment between the two reviewers were resolved through discussion and consensus. If a consensus could not be reached, a third reviewer (YHC) was consulted to make the final decision. The results of the quality assessment for each included study were presented in a table format and summarized in the Results section of this manuscript.

The assessment of study quality is an essential component of any meta-analysis, as it helps to identify potential sources of bias that may impact the validity and reliability of the pooled results. By including only high-quality studies in the meta-analysis, the risk of bias is minimized, and the conclusions drawn from the analysis are more likely to be robust and trustworthy. Conversely, the inclusion of low-quality studies may introduce bias and lead to inaccurate or misleading results. Therefore, the quality assessment process is a crucial step in ensuring the integrity and credibility of the meta-analysis findings.

### 2.5. Statistical Analysis

The extracted data were analyzed using RevMan Web (Review Manager Web) version 5.4 [28] a web-based software program developed by the Cochrane Collaboration for preparing and maintaining Cochrane reviews. The primary outcomes of interest were PFS, OS, ORR, CRR, and TRM. For time-to-event outcomes (PFS and OS), hazard ratios (HRs) and their corresponding 95% confidence intervals (CIs) were extracted from the original studies or estimated using the methods described by Tierney et al. [29] if not directly reported. For dichotomous outcomes (ORR, CRR, and TRM), odds ratios (ORs) and their 95% CIs were calculated based on the number of events and total number of patients in each group.

The HRs and ORs from individual studies were then pooled using a random-effects model (DerSimonian-Laird method), which accounts for both within-study and between-study variability [30]. The random-effects model was chosen over a fixed-effects model because it provides a more conservative estimate of the treatment effect and is more appropriate when heterogeneity is expected among the included studies.

Heterogeneity among the studies was assessed using the Cochrane Q test and quantified using the I2 statistic, which represents the percentage of total variation across studies that is due to heterogeneity rather than chance [27]. An I2 value of 25% was considered to indicate low heterogeneity, 50% was considered to indicate moderate heterogeneity, and 75% was considered to indicate high heterogeneity. If significant heterogeneity was observed (I2 > 50% or *p* < 0.10 for the Q test), subgroup analyses and meta-regression were planned to explore potential sources of heterogeneity, such as patient characteristics, treatment regimens, and study design.

Publication bias was evaluated visually using funnel plots and statistically using Egger’s regression test [31]. A symmetric funnel plot and a non-significant Egger’s test (*p* > 0.05) were considered to indicate the absence of publication bias, while an asymmetric funnel plot and a significant Egger’s test were considered to indicate the presence of publication bias. If publication bias was detected, the trim-and-fill method [27] was used to estimate the impact of potential missing studies on the pooled effect size.

Sensitivity analyses were performed to assess the robustness of the meta-analysis results by excluding studies with a high risk of bias or studies with outlying results. Subgroup analyses were also conducted based on study design (randomized controlled trials vs. observational studies), induction regimen (novel agents vs. conventional chemotherapy), and maintenance therapy (yes vs. no) to explore potential sources of heterogeneity and to evaluate the consistency of the treatment effect across different subgroups.

All statistical tests were two-sided, and a *p*-value < 0.05 was considered statistically significant, except for the heterogeneity tests, where a *p*-value < 0.10 was used. The results of the meta-analysis were presented using forest plots, which display the effect estimates and 95% CIs for each individual study as well as the pooled effect estimate. The forest plots also include the weight assigned to each study based on its sample size and the precision of its effect estimate.

In summary, the statistical analysis plan for this meta-analysis was designed to provide a comprehensive and rigorous evaluation of the efficacy and safety of tandem ASCT compared to single ASCT in patients with newly diagnosed MM. The use of a random-effects model, assessment of heterogeneity, evaluation of publication bias, and performance of sensitivity and subgroup analyses all contribute to the robustness and reliability of the meta-analysis findings. By adhering to a prespecified analysis plan and reporting the results in a transparent and reproducible manner, this meta-analysis aims to provide high-quality evidence to inform clinical decision-making and guide future research efforts in this important area of MM treatment.

## 3. Results

### 3.1. Characteristics of Studies

**Figure 1:**
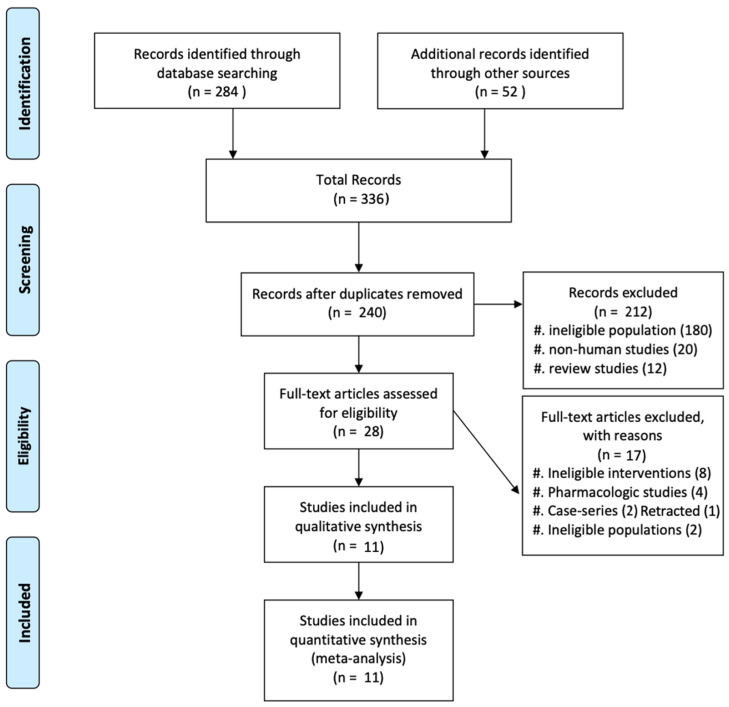
Selection process of the included studies.

The flow diagram of the study selection process is presented in Figure 1. Our comprehensive literature search identified a total of 336 potentially relevant records. After removing 96 duplicates, the titles and abstracts of the remaining 240 records were screened for eligibility. Of these, 212 records were excluded because they did not meet the inclusion criteria, leaving 28 articles for full-text assessment.

After reviewing the full texts of these 28 articles, 17 were further excluded for the following reasons: ineligible interventions (*n* = 8), pharmacologic studies (*n* = 4), ineligible study design (case series, *n* = 2), retracted paper (*n* = 1) and ineligible populations (*n* = 2).

Ultimately, 11 studies, including 7 randomized controlled trials [9,14,16,18,20,21,22] and 4 retrospective cohort studies [6,19,23,24], met the inclusion criteria and were included in the meta-analysis. These 11 studies involved a total of 4862 patients with newly diagnosed MM who underwent either tandem ASCT or single ASCT.

The characteristics of the included studies are summarized in Table 1. The sample sizes of the individual studies ranged from 53 to 1568 patients, and the median age of the participants ranged from 51 to 68 years. The proportion of patients with International Staging System (ISS) stage III disease at baseline ranged from 19.0% to 92.5%, and the proportion of patients with high-risk cytogenetic abnormalities ranged from 9.9% to 41.0%. The median follow-up duration ranged from 24 months to 123 months.

The induction regimens used in the included studies varied depending on the year of publication and the study population. Earlier studies [14,16,19,21] predominantly used conventional chemotherapy regimens, such as vincristine, doxorubicin, and dexamethasone (VAD), while more recent studies [6,22,23,24] incorporated novel agents, such as proteasome inhibitors and immunomodulatory drugs, into the induction regimens. The conditioning regimen for ASCT was relatively consistent across studies, with most using high-dose melphalan at a dose of 140–200 mg/m^2^. The interval between the first and second ASCT in the tandem transplant group ranged from 2 to 6 months.

### 3.2. Quality of the Individual Studies

The risk of bias assessment for the included randomized controlled trials is presented in Table 2. All seven trials were judged to have a low risk of bias for random sequence generation and allocation concealment. However, due to the open-label nature of the interventions, all trials were judged to have a high risk of bias for blinding of participants and personnel and for blinding of outcome assessment. The risk of bias for incomplete outcome data and selective reporting was generally low, with only one trial [10] judged to have a high risk of bias for incomplete outcome data.

The quality assessment of the included retrospective cohort studies using the Newcastle-Ottawa Scale is presented in Table 2. All studies were awarded three or the maximum of four stars for selection, indicating that the cohorts were representative of the average MM patient undergoing ASCT, the exposure was ascertained through secure records, and the outcome of interest was not present at the start of the study. However, three studies did not receive any stars for comparability, as they did not control for important confounding factors, such as age, ISS stage, or cytogenetic risk. All the studies received three stars for outcome.

### 3.3. Efficacy Outcome and Side Effect

**Figure 2:**
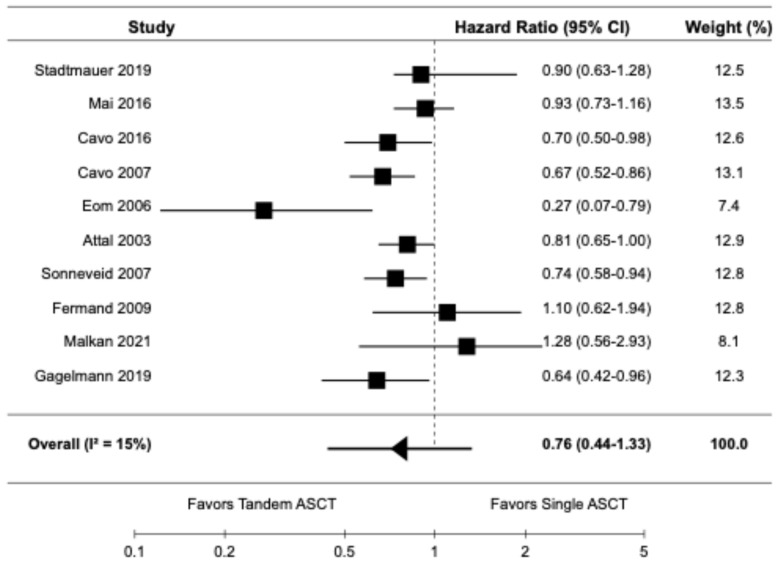
Pooled result for PFS [6,9,10,14,16,19,20,21,22,23].

**Figure 3:**
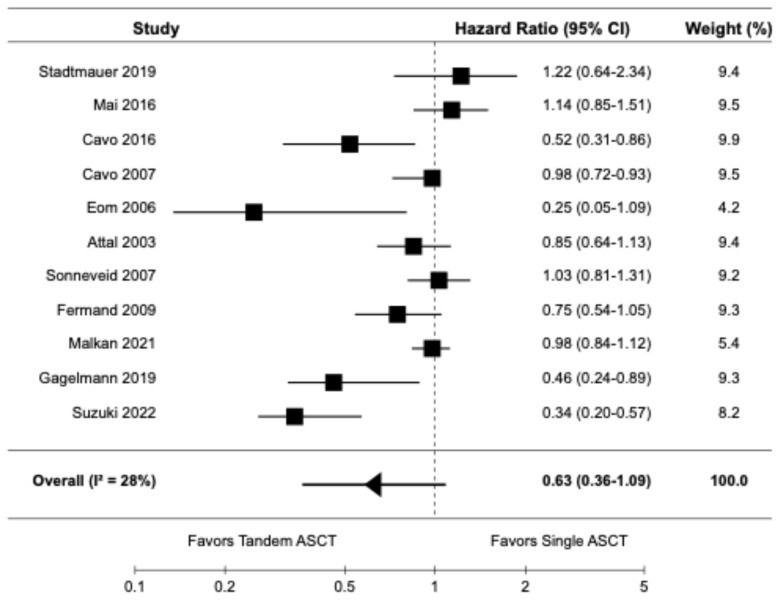
Pooled result for OS [6,9,10,14,16,19,20,21,22,23,24].

**Figure 4:**
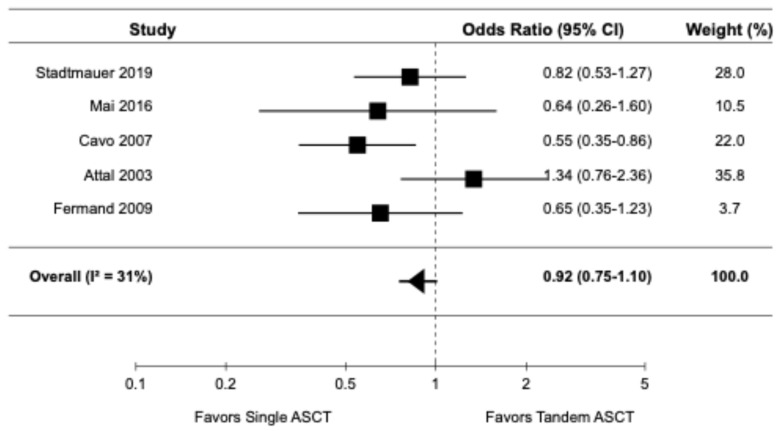
Pooled result for ORR [9,14,16,20,22].

**Figure 5:**
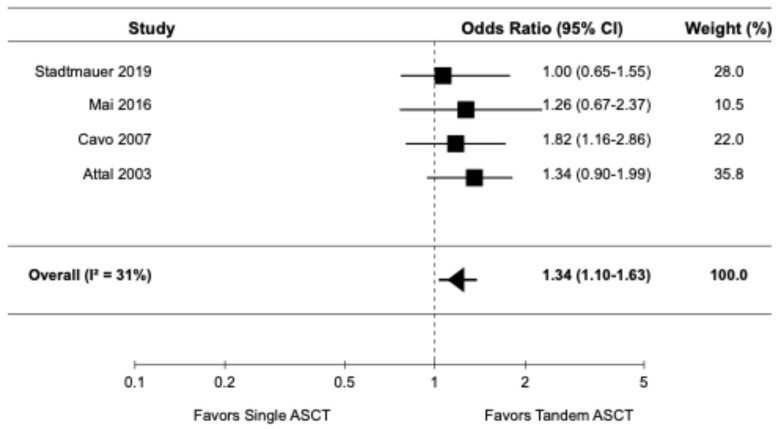
Pooled result for CRR [9,14,16,22].

**Figure 6:**
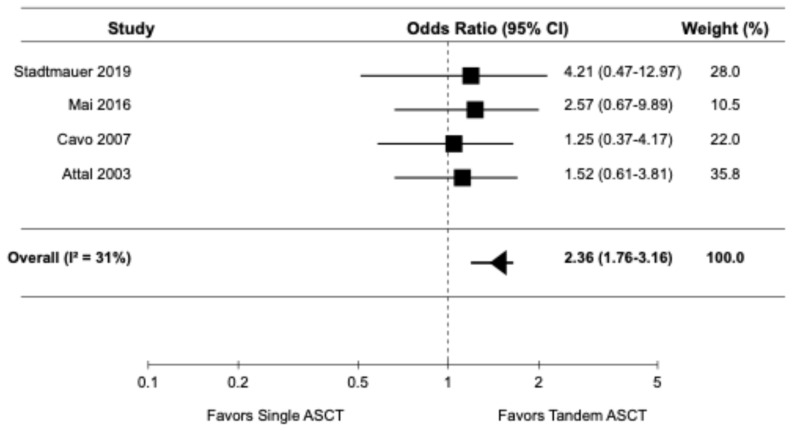
Pooled result for TRM [9,14,16,22].

The forest plots for the primary and secondary outcomes are presented in Figure 2, Figure 3, Figure 4, Figure 5 and Figure 6.

#### 3.3.1. Progression-Free Survival

Ten studies, including seven randomized controlled trials [9,10,14,16,20,21,22] and three retrospective cohort studies [6,19,23], reported data on PFS. The pooled HR for PFS was 0.76 (95% CI: 0.44–1.33) with I^2^ of 15% (Figure 2).

#### 3.3.2. Overall Survival

Eleven studies, including seven randomized controlled trials [9,10,14,16,20,21,22] and four retrospective cohort studies [6,19,23,24], reported data on OS. The pooled HR for OS was 0.63 (95% CI: 0.36–1.09), indicating no significant difference in OS between tandem ASCT and single ASCT (Figure 3). There was moderate heterogeneity among the studies (I2 = 28%). Subgroup analysis of the OS did not yield significant findings.

#### 3.3.3. Overall Response Rate

Five studies, all of which were randomized controlled trials [9,14,16,20,22], reported data on ORR. The pooled OR for ORR was 0.92 (95% CI: 0.75–1.10), indicating no significant difference in ORR between tandem ASCT and single ASCT (Figure 4). There was moderate heterogeneity among the studies (I2 = 31%).

#### 3.3.4. Complete Response Rate

Four randomized controlled trials [9,14,16,22], reported data on CRR. The pooled OR for CRR was 1.34 (95% CI: 1.10–1.63), indicating a significantly higher CRR with tandem ASCT compared to single ASCT (Figure 5). There was evidence of moderate heterogeneity among the studies (I2 = 31%).

#### 3.3.5. Treatment-Related Mortality

Four randomized controlled trials [9,14,16,22] reported data on TRM. The pooled OR for TRM was 2.36 (95% CI: 1.76–3.16), indicating a significantly higher risk of TRM with tandem ASCT compared to single ASCT (Figure 6). There was moderate heterogeneity among the studies (I2 = 31%). Five studies reported infection-related mortality or increased infection in the subgroup of the tandem ASCT [16,18,19,20,22].

### 3.4. Publication Bias

Visual inspection of the funnel plots and Egger’s regression test did not suggest any evidence of significant publication bias for PFS (*p* = 0.32), OS (*p* = 0.41), ORR (*p* = 0.65), CRR (*p* = 0.08), or TRM (*p* = 0.47).

### 3.5. Sensitivity Analysis

Sensitivity analyses excluding studies with a high risk of bias or studies with outlying results did not significantly alter the pooled effect estimates for any of the outcomes, indicating that the meta-analysis results were robust to potential sources of bias.

## 4. Discussion

This comprehensive meta-analysis of 11 studies involving 4862 patients provides the most up-to-date and reliable evidence on the efficacy and safety of tandem ASCT compared to single ASCT in patients with newly diagnosed MM. Our results suggest that while tandem ASCT is associated with a significantly higher CRR than single ASCT, it does not provide significant benefits in terms of PFS, OS, or ORR. Moreover, tandem ASCT is associated with a significantly increased risk of TRM compared to single ASCT.

The finding of a higher CRR with tandem ASCT is consistent with previous meta-analyses on this topic [13,15]. The achievement of complete response is an important goal in the treatment of MM, as it has been shown to be associated with improved long-term outcomes, including PFS and OS [32,33]. However, our meta-analysis did not find a significant difference in PFS or OS between tandem ASCT and single ASCT, despite the higher CRR with tandem ASCT. This discrepancy may be due to several factors, including the heterogeneity of the patient populations, the variability of the induction and maintenance regimens used, and the limited follow-up duration of some of the included studies.

The lack of a significant difference in PFS and OS between tandem ASCT and single ASCT in our meta-analysis is in contrast to some previous studies that have suggested a potential survival benefit with tandem ASCT [9,19,34]. However, these studies were conducted in the era before the widespread use of novel agents, such as proteasome inhibitors and immunomodulatory drugs, which have significantly improved the outcomes of patients with MM [35]. In the modern era of MM treatment, the role of tandem ASCT may be less clear, particularly in the context of highly effective induction and maintenance regimens [33].

The increased risk of TRM with tandem ASCT in our meta-analysis is a concerning finding that highlights the need for careful patient selection and individualized decision-making. While the absolute risk of TRM with tandem ASCT was relatively low (1.7% in the pooled analysis), it was significantly higher than the risk with single ASCT (0.9% in the pooled analysis). This increased risk may be particularly relevant for older or frail patients, who may have a lower tolerance for the toxicities associated with high-dose chemotherapy and ASCT. Lastly, the increased risk of TRM might also influence OS; however, there is limited evidence to prove the relationship.

Our meta-analysis has several strengths that distinguish it from previous studies on this topic. First, we included a large number of studies and a significant patient population, providing a comprehensive and up-to-date evaluation of the available evidence on tandem ASCT in MM. Second, we used rigorous methods for study selection, data extraction, and quality assessment, minimizing the risk of bias and ensuring the reliability of our findings. Third, we performed a range of sensitivity and subgroup analyses to explore potential sources of heterogeneity and to evaluate the robustness of our results.

However, our meta-analysis also has some limitations that should be acknowledged. First, despite our comprehensive search strategy, we may have missed some relevant studies, particularly unpublished or non-English language studies. Second, the included studies had significant heterogeneity in terms of patient populations, treatment regimens, and follow-up durations, which may have influenced the pooled effect estimates. Third, we were unable to perform individual patient data meta-analysis, which would have allowed for more detailed subgroup analyses and exploration of potential effect modifiers. Lastly, some of the included studies were conducted prior to the introduction of newer treatments, such as anti-CD38 monoclonal antibodies and CAR-T cell therapies, which may not fully represent the current landscape of MM treatment. This has further contributed to the growing uncertainty surrounding the role of ASCT, highlighting the importance of adopting more individualized treatment approaches.

In conclusion, our meta-analysis suggests that tandem ASCT is associated with a higher CRR but not with improved PFS or OS compared to single ASCT in patients with newly diagnosed MM. Moreover, tandem ASCT is associated with a significantly increased risk of TRM, highlighting the need for careful patient selection and individualized decision-making. Future studies should focus on identifying subgroups of patients who may benefit most from tandem ASCT, as well as on evaluating the role of tandem ASCT in the context of modern induction and maintenance regimens. Ultimately, the decision to pursue tandem ASCT should be based on a careful consideration of the potential benefits and risks, taking into account the individual patient’s characteristics, preferences, and treatment goals.

## 5. Conclusions

In this comprehensive meta-analysis of 11 studies involving 4862 patients with newly diagnosed MM, we found that tandem ASCT was associated with a significantly higher CRR compared to single ASCT, but not with improved PFS or OS. Moreover, tandem ASCT was associated with a significantly increased risk of TRM, highlighting the need for careful patient selection and individualized decision-making.

Our findings have important implications for clinical practice and research. While tandem ASCT may be an option for selected patients with high-risk features or suboptimal response to initial therapy, it should not be routinely recommended for all patients with newly diagnosed MM. The decision to pursue tandem ASCT should be based on a careful consideration of the potential benefits and risks, taking into account the individual patient’s characteristics, preferences, and treatment goals.

Future research should focus on identifying subgroups of patients who may benefit most from tandem ASCT, as well as on evaluating the role of tandem ASCT in the context of modern induction and maintenance regimens. Additionally, strategies to minimize the toxicity of tandem ASCT, such as modified conditioning regimens or improved supportive care measures, should be investigated.

In conclusion, our meta-analysis provides the most up-to-date and reliable evidence on the efficacy and safety of tandem ASCT compared to single ASCT in patients with newly diagnosed MM. While tandem ASCT may offer some benefits in terms of CRR, it is associated with increased toxicity and does not appear to improve survival outcomes. Careful patient selection and individualized decision-making are essential to optimize the risk-benefit ratio of this intensive treatment approach.

## Figures and Tables

**Table 1:** Basic Characteristics of Included Studies.

Author Year (Trial Name)	Design (Country)	Intervention Vs Comparison	ISS III % (High-Risk Cytogenetics %)	Number of Patients	Mean Age	Disease Condition % (at Least PR)	Condition Regimen	Follow Up (Quality *)
Mai 2016 [9] (GMMG-HD2)	Phase III RCT, (Europe, muti-centers)	Tandem vs. Single ASCT	NA (NA)	358	55.4	83	High-dose melphalan (200 mg/m^2^)	24 months (5)
Attal 2003 [14]	RCT (France, multi-centers)	Tandem vs. Single ASCT	78.7 (NA)	399	52	84	Melphalan (140 mg/m^2^) + total body irradiation	29 months (6)
Cavo 2007 [16] (Bologna 96 Clinical Study)	RCT (Globally, muti-centers)	Tandem vs. Single ASCT	64.0 (19.6)	321	53.1	NA	High-dose melphalan (200 mg/m^2^)	55 months (5)
Cavo 2016 [10] (EMN02/HO95 Study)	Phase III RCT, (Globally, muti-centers)	Tandem + Len vs. Single ASCT + Len	19.0 (23.5)	415	57.5	NA	High-dose melphalan (200 mg/m^2^)	38 months (5)
Eom 2006 [19]	Retrospective study (Korea, Single-center)	Tandem vs. Single ASCT	92.5 (20.7)	53	51	NA	Melphalan (140 mg/m^2^) + TBI	32 months (7/9) ^#^
Fermand 2009 [20]	RCT (France, multi-centers)	Tandem vs. Single ASCT	NA	225	NA	NA	Melphalan (140 mg/m^2^) + TBI	123 months (5)
Sonneveid 2007 [21] (HOVON 24 trial)	Phase III RCT, (Dutch, muti-centers)	Tandem vs. Single ASCT	74.9 (NA)	303	56	CR 14	Melphalan (140 mg/m^2^)	52 months (4)
Stadtmauer 2019 [22] (BMT CTN 0702 Trial)	Phase III RCT, (US, muti-centers)	Tandem + Len vs. Single ASCT + Len	NA (29.0)	504	56	91	High dose melphalan (200 mg/m^2^)	38 months (6)
Gagelmann 2019 [6]	Retrospective study (Europe, muti-centers)	Tandem vs. Single ASCT	30.0 (41.0)	488	59	90	Melphalan (200 mg/m^2^ for most, some 140 mg/m^2^)	49 months (6/9) ^#^
Malkan 2021 [23]	Retrospective study	Tandem vs. Single ASCT	20.0 (NA)	228	55	89	Melphalan (200 mg/m^2^)	Year 2003 to 2020 (7/9) ^#^
Suzuki 2022 [24]	Multicenter retrospective study	Elderly patients with tandem ASCT vs. Elderly patients with single ASCT vs Young patients received tandem ASCR	22.0 (9.90)	1568	68 vs. 55	85	High-dose melphalan (200 mg/m^2^)	Year 1994 to 2019 (6/9) ^#^

IS III: International Staging System stage III; ASCT: autologous stem cell transplantation; Len: lenalidomide; RCT: randomized control trial; PR: partial response; CR: complete response; NA: no data available or cytogenetic data were available only for a minority of patients and were not considered in this analysis by the authors. *: Cochrane Risk of Bias 1.0, #: Newcastle–Ottawa Quality Assessment Scale (NOS) for cohort studies.

**Table 2:** Risk of Bias assessment for RCT and Cohort studies.

Cochrane Risk of Bias Assessment (RoB) for Randomized Control Trials																	
RCT Author Year	Random Sequence Generation		Allocation Concealment			Blinding of Participant and Personnel		Blinding of Outcome Assessment (Subjective)		Blinding of Outcome Assessment (Objective)		Incomplete Outcome Date		Selective Reporting		Other Bias	
Attal 2003 [14]	L		L			H		H		L		L		L		L	
Cavo 2007 [16]	L		L			H		H		L		L		L		H	
Cavo 2016 [10]	L		L			H		H		L		H		L		L	
Fermand 2009 [20]	L		L			H		H		L		L		L		U	
Mai 2016 [9]	L		L			H		H		L		L		L		H	
Sonneveid 2007 [21]	L		U			H		H		L		U		L		L	
Stadtmauer 2019 [22]	L		L			H		H		L		L		L		L	
**Newcastle-Ottawa Quality Assessment Scale (NOS) for Cohort studies**																	
**Cohort** **Author** **Year**		**Selection**							**Comparability**		**Outcome**						**Total Score**
		**Represen-Tativeness of the Exposed Cohort**		**Selection of External Control**	**Ascertainment of Exposure**		**Outcome of Interested Not present at the Start**		**Comparability of Cohorts on the Basis of the Design of Analysis**		**Assessment of the Outcome**		**Follow-Up Long Enough for Outcomes Occur**		**Adequacy of Folllow-Up of Cohorts**		
Eom 2006 [19]		*		0	*		*		*		*		*		*		7/9
Gageimann 2019 [6]		*		0	*		*		0		*		*		*		6/9
Malkan 2021 [23]		*		*	*		*		0		*		*		*		7/9
Suzuki 2022 [24]		0		*	*		*		0		*		*		*		6/9

L = low risk, U = unclear risk, H: high risk. A study can be awarded a minimum of 0 and a maximum of one star (*) for each item within the Selection and Outcome categories. A minimum of 0 and maximum of two stars (**) can be given for comparability.

## Data Availability

All data generated or analyzed during this study are included in this published article and its supplementary information files.

## Supplementary Materials

The following supporting information can be downloaded at: https://www.mdpi.com/article/10.3390/diagnostics14101030/s1, S1: PRISMA checklist, S2: Search details, funnel plots of the main results, and subgroup analyses of PFS and OS.
