# Correction: Chen et al. The Efficacy and Safety of Tandem Transplant Versus Single Stem Cell Transplant for Multiple Myeloma Patients: A Systematic Review and Meta-Analysis. *Diagnostics* 2024, *14*, 1030

**DOI:** 10.3390/diagnostics15232942

**Published:** 2025-11-21

**Authors:** Yu-Han Chen, Lindsay Fogel, Andrea Yue-En Sun, Chieh Yang, Rushin Patel, Wei-Cheng Chang, Po-Huang Chen, Hong-Jie Jhou, Yeu-Chin Chen, Ming-Shen Dai, Cho-Hao Lee

**Affiliations:** 1 Department of Internal Medicine, Englewood Hospital and Medical Center, Englewood, NJ 07631, USA; yuhanchen002@gmail.com; 2 Hackensack Meridian School of Medicine, Nutley, NJ 07110, USA; lindsay.fogel@hmhn.org; 3 College of Medicine, National Yang Ming Chiao Tung University, Taipei 112, Taiwan; andreaysun@gmail.com; 4 Department of Internal Medicine, School of Medicine, University of California Riverside, Riverside, CA 92521, USA; chiehy@medsch.ucr.edu; 5 Department of Internal Medicine, Community Hospital of San Bernardino, San Bernardino, CA 92411, USA; rushinpateldr@gmail.com; 6 Department of Ophthalmology, Taoyuan General Hospital, Ministry of Health and Welfare, Taoyuan 330, Taiwan; cwc761229@gmail.com; 7 Department of Internal Medicine, Tri-Service General Hospital, National Defense Medical Center, Taipei 114, Taiwan; chenpohuang@hotmail.com; 8 Department of Neurology, Changhua Christian Hospital, Changhua 500, Taiwan; xsai4295@gmail.com; 9 Division of Hematology and Oncology Medicine, Department of Internal Medicine, Tri-Service General Hospital, National Defense Medical Center, Taipei 114, Taiwan; yeuchin99@gmail.com (Y.-C.C.); dms1201@gmail.com (M.-S.D.)

After publication, the authors identified several errors in the manuscript, including inconsistencies between the manuscript’s reported results and the corresponding figures, a missing citation, and incorrect reference citations. The concerns raised by the reviewers for this correction were also addressed, including the types of the article included and cited, the addition of limitations, language corrections, and removal of one retracted reference.

It should be noted that the original figures accurately represent the original analysis. The discrepancies arose due to errors in the manuscript text, which did not accurately reflect the original findings. The changes were only made to remove the retracted study from the analysis.

The authors sincerely apologize for these mistakes and confirm that the scientific conclusions of the paper remain unaffected. These corrections have been approved by the Academic Editor, and the original publication has been updated to address these issues [1].

**Abstract and Methods Correction**

There was an error in the abstract and methods of the original publication. For the overall response rate (ORR), complete response rate (CRR), and treatment-related mortality (TRM) result, we used odds ratio (OR) instead of relative risk (RR) in the original analysis. The original figures’ results were not changed, but there were mismatches of the results in the manuscript text. Additionally, one study (Abdelkefi 2008) [2] was excluded from the meta-analysis because the single-ASCT arm included thalidomide maintenance therapy, introducing confounding not present in other studies. Furthermore, this study was retracted due to inappropriately collected initial data at the time of publication. To maintain the rigor and integrity of the analysis, the authors decided to exclude this study in this correction.

The following three places were corrected accordingly:

(1) A correction has been made to the Abstract, Methods:

We used a random-effects model to calculate pooled hazard ratios (HRs) and odds ratios (ORs) with 95% confidence intervals (CIs).

(2) A correction has been made to the Abstract, Result:

Eleven studies involving 4862 patients met the inclusion criteria. Tandem ASCT was associated with a significantly higher CRR compared to single ASCT (OR 1.34, 95% CI 1.10–1.63, I^2^ = 31%), but no significant differences were observed in PFS (HR 0.76, 95% CI 0.44–1.33, I^2^ = 15%), OS (HR 0.63, 95% CI 0.36–1.09, I^2^ = 28%), or the ORR (OR 0.92, 95% CI 0.75–1.10, I^2^ = 31%). However, tandem ASCT was associated with a significantly higher risk of TRM (OR 2.36, 95% CI 1.76–3.16, I^2^ = 31%).

(3) A correction has been made to the Materials and Methods, 2.2. Inclusion and exclusion criteria, Paragraph 2:

(4) studies with incomplete, retracted or duplicated data;

(4) A correction has been made to the Materials and Methods, 2.5. Statistical analysis, Paragraph 1 and Paragraph 2:

For dichotomous outcomes (ORR, CRR, and TRM), odds ratios (ORs) and their 95% CIs were calculated based on the number of events and total number of patients in each group.

The HRs and ORs from individual studies were then pooled using a random-effects model (DerSimonian-Laird method), which accounts for both within-study and between-study variability [30].

**Missing Citation**

In the original publication, Tierney, J.F.; Stewart, L.A.; Ghersi, D.; Burdett, S.; Sydes, M.R. Practical methods for incorporating summary time-to-event data into meta-analysis. *Trials* **2007**, *8*, 16, was not cited. The citation has now been inserted in 2.5. Statistical Analysis, Paragraph 1, and should read:

For time-to-event outcomes (PFS and OS), hazard ratios (HRs) and their corresponding 95% confidence intervals (CIs) were extracted from the original studies or estimated using the methods described by Tierney et al. [29] if not directly reported. For dichotomous outcomes (ORR, CRR, and TRM), odds ratios (ORs) and their 95% CIs were calculated based on the number of events and total number of patients in each group.

**Introduction Correction**

There was an error in the introduction of the original publication. It has been brought to our attention by the post-publication reviewer that the cited study was not a meta-analysis, but we mistakenly wrote it as a meta-analysis. A correction has been made to the Introduction, Paragraph 8, by removing the descriptor “meta-analyses”:

While previous studies have been conducted on this topic, they have several limitations, such as the inclusion of a small number of trials, lack of subgroup analyses, and failure to account for the quality of included studies [23,24].

**Results Correction**

There was an error in the result of the original publication. We used OR in our original analysis of ORR, CRR, and TRM, which was not correctly indicated in the manuscript. There was also correlated result changed due to the removal of the retracted study from the analysis. Six places were influenced and corrected as below. In addition, reference 20 was misidentified as a randomized controlled trial instead of a retrospective cohort study. The corresponding sentences have been revised accordingly.

(1) A correction has been made to Result, 3.1. Characteristics of Studies, Paragraph 1–4:

The flow diagram of the study selection process is presented in Figure 1. Our comprehensive literature search identified a total of 336 potentially relevant records. After removing 96 duplicates, the titles and abstracts of the remaining 240 records were screened for eligibility. Of these, 212 records were excluded because they did not meet the inclusion criteria, leaving 28 articles for full-text assessment.

After reviewing the full texts of these 28 articles, 17 were further excluded for the following reasons: ineligible interventions (*n* = 8), pharmacologic studies (*n* = 4), ineligible study design (case series, *n* = 2), retracted paper (*n* = 1) and ineligible populations (*n* = 2).

Ultimately, 11 studies, including 7 randomized controlled trials [9,14,16,18,20–22] and 4 retrospective cohort studies [6,19,23,24], met the inclusion criteria and were included in the meta-analysis. These 11 studies involved a total of 4862 patients with newly diagnosed MM who underwent either tandem ASCT or single ASCT.

The characteristics of the included studies are summarized in Table 1. The sample sizes of the individual studies ranged from 53 to 1568 patients, and the median age of the participants ranged from 51 to 68 years. The proportion of patients with International Staging System (ISS) stage III disease at baseline ranged from 19.0% to 92.5%, and the proportion of patients with high-risk cytogenetic abnormalities ranged from 9.9% to 41.0%.

(2) A correction has been made to Result, 3.3.1. Progression-Free Survival:

Ten studies, including seven randomized controlled trials [9,10,14,16,20–22] and three retrospective cohort studies [6,19,23], reported data on PFS. The pooled HR for PFS was 0.76 (95% CI: 0.44–1.33) with I^2^ of 15% (Figure 2).

(3) A correction has been made to Result, 3.3.2. Overall Survival:

Eleven studies, including seven randomized controlled trials [9,10,14,16,20–22] and four retrospective cohort studies [6,19,23,24], reported data on OS. The pooled HR for OS was 0.63 (95% CI: 0.36–1.09), indicating no significant difference in OS between tandem ASCT and single ASCT (Figure 3). There was moderate heterogeneity among the studies (I^2^ = 28%).

(4) A correction has been made to Result, 3.3.3. Overall Response Rate:

Five studies, all of which were randomized controlled trials [9,14,16,20,22], reported data on ORR. The pooled OR for ORR was 0.92 (95% CI: 0.75–1.10), indicating no significant difference in ORR between tandem ASCT and single ASCT (Figure 4). There was moderate heterogeneity among the studies (I^2^ = 31%).

(5) A correction has been made to Result, 3.3.4. Complete Response Rate:

Four randomized controlled trials [9,14,16,22], reported data on CRR. The pooled OR for CRR was 1.34 (95% CI: 1.10–1.63), indicating a significantly higher CRR with tandem ASCT compared to single ASCT (Figure 5). There was evidence of moderate heterogeneity among the studies (I^2^ = 31%).

(6) A correction has been made to Result, 3.3.5. Treatment-Related Mortality

Four randomized controlled trials [9,14,16,22] reported data on TRM. The pooled OR for TRM was 2.36 (95% CI: 1.76–3.16), indicating a significantly higher risk of TRM with tandem ASCT compared to single ASCT (Figure 6). There was moderate heterogeneity among the studies (I^2^ = 31%). Five studies reported infection-related mortality or increased infection in the subgroup of the tandem ASCT [16,18–20,22].

**Table Correction**

The retracted study (Abdelkefi 2008) was removed and tables were corrected accordingly. 

The study by Eom et al. (reference 20) was misidentified as a randomized controlled trial instead of a retrospective cohort study. Table 1 has been corrected to mark the Eom 2006 study as a retrospective study. Table 2 has also been corrected to change Eom 2006 to Newcastle–Ottawa Quality Assessment Scale (NOS) for Cohort studies, with a total score of 7/9. The corresponding texts in the manuscript were also corrected.

A correction has been made to the Results, 3.2. Quality of the Individual Studies, Paragraph 1 and Paragraph 2:

All seven trials were judged to have a low risk of bias for random sequence generation and allocation concealment.

All studies were awarded three or the maximum of four stars for selection, indicating that the cohorts were representative of the average MM patient undergoing ASCT, the exposure was ascertained through secure records, and the outcome of interest was not present at the start of the study. However, three studies did not receive any stars for comparability, as they did not control for important confounding factors, such as age, ISS stage, or cytogenetic risk. All the studies received three stars for outcome.

There was an error in Table 1, where “prospective” was mistakenly written as “perspective.” To ensure clarity and consistency throughout the manuscript, we have corrected this and referred to it simply as an RCT in Table 1. The correct Table 1 and Table 2 are as follows.

**Table 1:** Basic Characteristics of Included Studies.

Author Year (Trial Name)	Design (Country)	Intervention Vs Comparison	ISS III % (High-Risk Cytogenetics %)	Number of Patients	Mean Age	Disease Condition % (at Least PR)	Condition Regimen	Follow Up (Quality *)
Mai 2016 [9] (GMMG-HD2)	Phase III RCT, (Europe, muti-centers)	Tandem vs. Single ASCT	NA (NA)	358	55.4	83	High-dose melphalan (200 mg/m^2^)	24 months (5)
Attal 2003 [14]	RCT (France, multi-centers)	Tandem vs. Single ASCT	78.7 (NA)	399	52	84	Melphalan (140 mg/m^2^) + total body irradiation	29 months (6)
Cavo 2007 [16] (Bologna 96 Clinical Study)	RCT (Globally, muti-centers)	Tandem vs. Single ASCT	64.0 (19.6)	321	53.1	NA	High-dose melphalan (200 mg/m^2^)	55 months (5)
Cavo 2016 [10] (EMN02/HO95 Study)	Phase III RCT, (Globally, muti-centers)	Tandem + Len vs. Single ASCT + Len	19.0 (23.5)	415	57.5	NA	High-dose melphalan (200 mg/m^2^)	38 months (5)
Eom 2006 [19]	Retrospective study (Korea, Single-center)	Tandem vs. Single ASCT	92.5 (20.7)	53	51	NA	Melphalan (140 mg/m^2^) + TBI	32 months (7/9) ^#^
Fermand 2009 [20]	RCT (France, multi-centers)	Tandem vs. Single ASCT	NA	225	NA	NA	Melphalan (140 mg/m^2^) + TBI	123 months (5)
Sonneveid 2007 [21] (HOVON 24 trial)	Phase III RCT, (Dutch, muti-centers)	Tandem vs. Single ASCT	74.9 (NA)	303	56	CR 14	Melphalan (140 mg/m^2^)	52 months (4)
Stadtmauer 2019 [22] (BMT CTN 0702 Trial)	Phase III RCT, (US, muti-centers)	Tandem + Len vs. Single ASCT + Len	NA (29.0)	504	56	91	High dose melphalan (200 mg/m^2^)	38 months (6)
Gagelmann 2019 [6]	Retrospective study (Europe, muti-centers)	Tandem vs. Single ASCT	30.0 (41.0)	488	59	90	Melphalan (200 mg/m^2^ for most, some 140 mg/m^2^)	49 months (6/9) ^#^
Malkan 2021 [23]	Retrospective study	Tandem vs. Single ASCT	20.0 (NA)	228	55	89	Melphalan (200 mg/m^2^)	Year 2003 to 2020 (7/9) ^#^
Suzuki 2022 [24]	Multicenter retrospective study	Elderly patients with tandem ASCT vs. Elderly patients with single ASCT vs Young patients received tandem ASCR	22.0 (9.90)	1568	68 vs. 55	85	High-dose melphalan (200 mg/m^2^)	Year 1994 to 2019 (6/9) ^#^

IS III: International Staging System stage III; ASCT: autologous stem cell transplantation; Len: lenalidomide; RCT: randomized control trial; PR: partial response; CR: complete response; NA: no data available or cytogenetic data were available only for a minority of patients and were not considered in this analysis by the authors. *: Cochrane Risk of Bias 1.0, #: Newcastle–Ottawa Quality Assessment Scale (NOS) for cohort studies.

**Table 2:** Risk of Bias assessment for RCT and Cohort studies.

Cochrane Risk of Bias Assessment (RoB) for Randomized Control Trials									
RCT Author Year	Random Sequence Generation	Allocation Concealment	Blinding of Participant and Personnel	Blinding of Outcome Assessment (Subjective)	Blinding of Outcome Assessment (Objective)	Incomplete Outcome Date	Selective Reporting		Other Bias
Attal 2003 [14]	L	L	H	H	L	L	L		L
Cavo 2007 [16]	L	L	H	H	L	L	L		H
Cavo 2016 [10]	L	L	H	H	L	H	L		L
Fermand 2009 [20]	L	L	H	H	L	L	L		U
Mai 2016 [9]	L	L	H	H	L	L	L		H
Sonneveid 2007 [21]	L	U	H	H	L	U	L		L
Stadtmauer 2019 [22]	L	L	H	H	L	L	L		L
**Newcastle-Ottawa Quality Assessment Scale (NOS) for Cohort studies**									
**Cohort** **Author** **Year**	**Selection**				**Comparability**	**Outcome**			**Total Score**
	**Represen-Tativeness of the Exposed Cohort**	**Selection of External Control**	**Ascertainment of Exposure**	**Outcome of Interested Not present at the Start**	**Comparability of Cohorts on the Basis of the Design of Analysis**	**Assessment of the Outcome**	**Follow-Up Long Enough for Outcomes Occur**	**Adequacy of Folllow-Up of Cohorts**	
Eom 2006 [19]	*	0	*	*	*	*	*	*	7/9
Gageimann 2019 [6]	*	0	*	*	0	*	*	*	6/9
Malkan 2021 [23]	*	*	*	*	0	*	*	*	7/9
Suzuki 2022 [24]	0	*	*	*	0	*	*	*	6/9

L = low risk, U = unclear risk, H: high risk. A study can be awarded a minimum of 0 and a maximum of one star (*) for each item within the Selection and Outcome categories. A minimum of 0 and maximum of two stars (**) can be given for comparability.

**Figure Correction**

The retracted study (Abdelkefi 2008) was removed from the analysis and figures have been updated accordingly. The scientific conclusion of this study remains unchanged.

**Figure 1:**
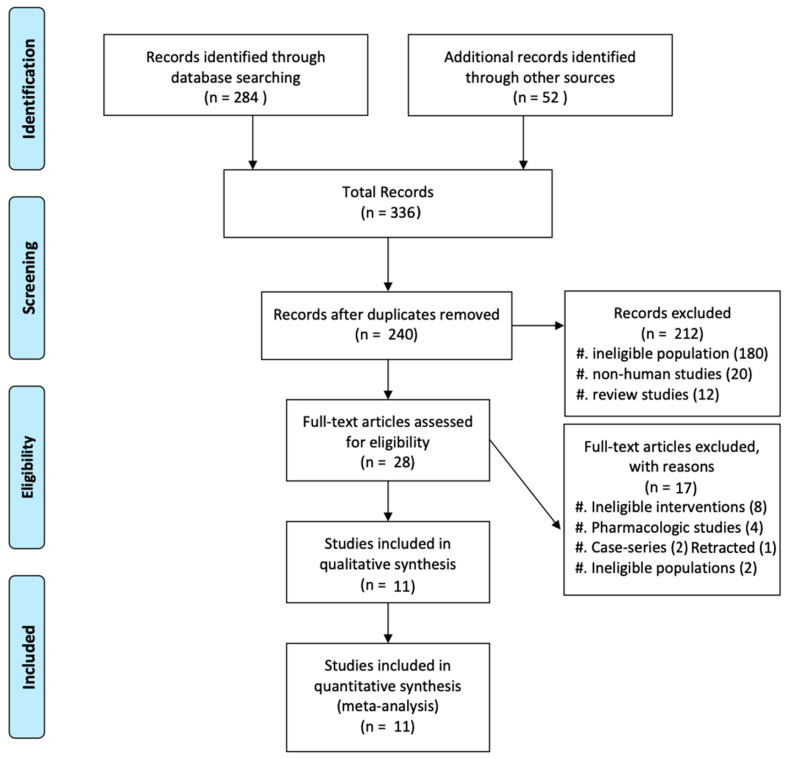
Selection process of the included studies.

**Figure 2:**
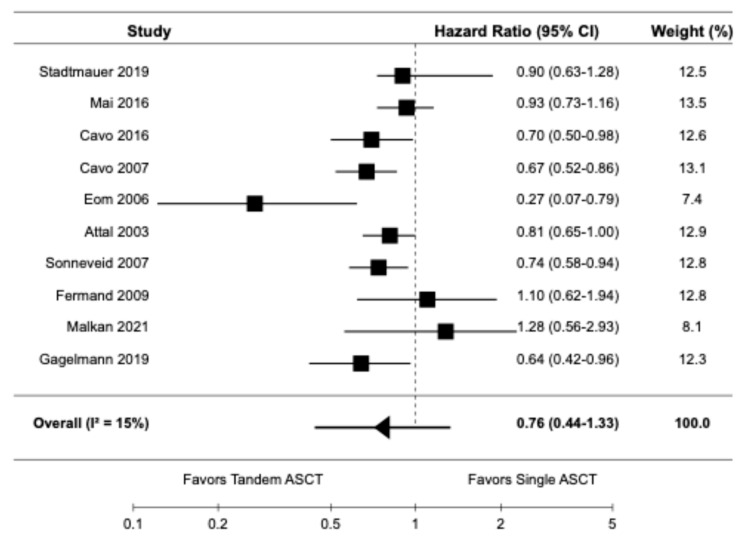
Pooled result for PFS [6,9,10,14,16,19–23].

**Figure 3:**
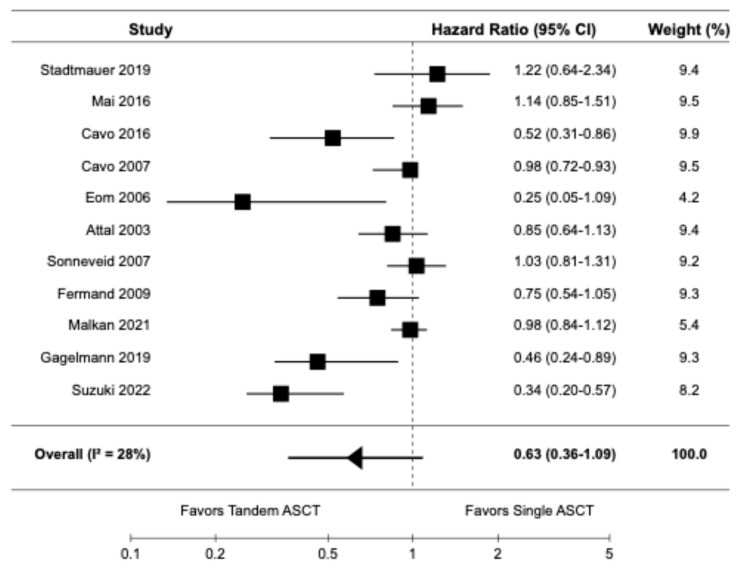
Pooled result for OS [6,9,10,14,16,19–24].

**Figure 4:**
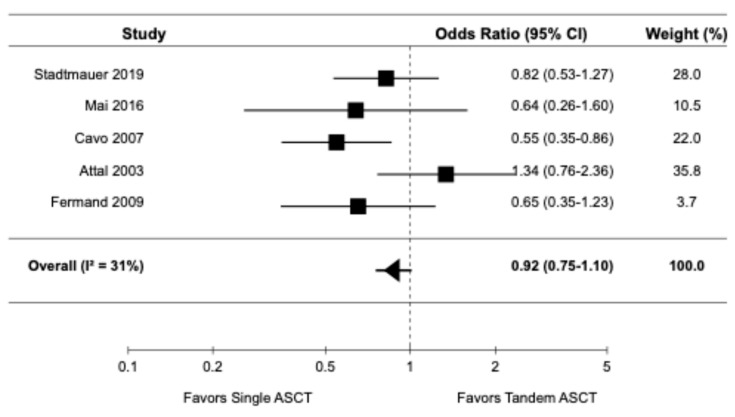
Pooled result for ORR [9,14,16,20,22].

**Figure 5:**
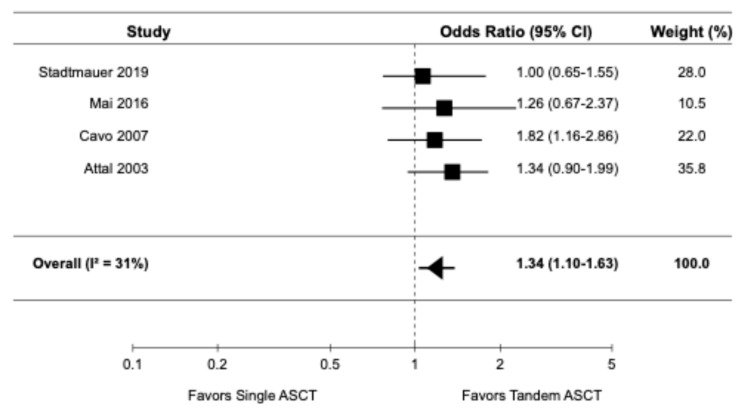
Pooled result for CRR [9,14,16,22].

**Figure 6:**
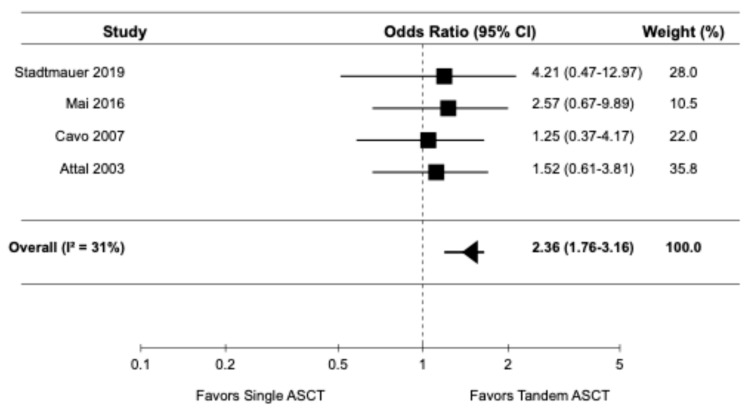
Pooled result for TRM [9,14,16,22].

**Discussion and Conclusion Correction**

Corrections were made to reflect the removal of one of the studies.

(1) Discussion, Paragraph 1:

This comprehensive meta-analysis of 11 studies involving 4862 patients provides the most up-to-date and reliable evidence on the efficacy and safety of tandem ASCT compared to single ASCT in patients with newly diagnosed MM.

(2) Conclusions, Paragraph 1

In this comprehensive meta-analysis of 11 studies involving 4862 patients with newly diagnosed MM, we found that tandem ASCT was associated with a significantly higher CRR compared to single ASCT, but not with improved PFS or OS.

The post-publication reviewer expressed concern regarding the limitation of the original publication. The included studies, conducted many years ago, may not reflect modern myeloma treatment at best. A correction has been made to Discussion, Paragraph 6:

Lastly, some of the included studies were conducted prior to the introduction of newer treatments, such as anti-CD38 monoclonal antibodies and CAR-T cell therapies, which may not fully represent the current landscape of MM treatment. This has further contributed to the growing uncertainty surrounding the role of ASCT, highlighting the importance of adopting more individualized treatment approaches.

**Language Correction**

The language was carefully reviewed to correct typographical errors and ensure the proper use of abbreviations.

**References Correction**

There was an error in the order of the references in the original publication. With this correction, the order of some references has been adjusted accordingly.
